# Supplementary material for: Immunoinformatic Execution and Design of an Anti-Epstein–Barr Virus Vaccine with Multiple Epitopes Triggering Innate and Adaptive Immune Responses
Source: Microorganisms. 2023 Sep 29;11(10):2448. doi: 10.3390/microorganisms11102448 (PMC10609278; doi:10.3390/microorganisms11102448)
Supplement: Supplementary file 1 [file microorganisms-11-02448-s001.zip › microorganisms-2570497-supplementary.pdf]

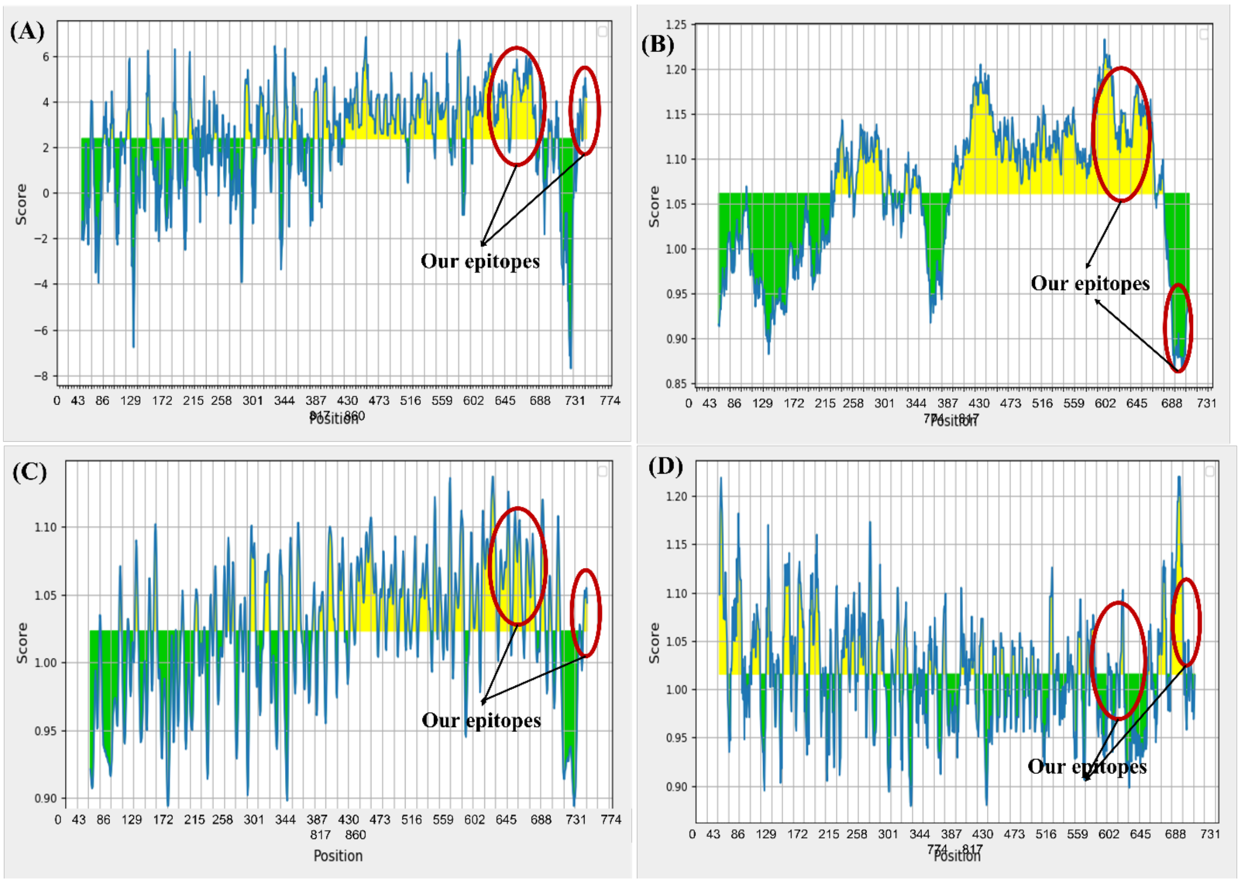

Figure S1: Graphical representation of the immunogenic epitopes. (A) B-turn prediction; (B) Surface accessibility; (C) Flexibility; (D) Antigenicity.

(A)

Query= FVC2

Length=386

Sequences producing significant alignments:	Score (Bits)	E Value
drugbank_target|P03200 Envelope glycoprotein GP350 (DB00632)	128	1e-032
drugbank_target|P68344 Envelope glycoprotein GP340 (DB00632)	127	3e-032
T23459|VGP3_EBVP3	127	3e-032
T69200|SMCA4_HUMAN	29.3	2.3
drugbank_target|Q8IC05 Heat shock protein 90 (DB11638)	28.9	2.6
drugbank_target|Q04609 Glutamate carboxypeptidase 2 (DB00089; D...	28.1	4.4
T97071|FOLH1_HUMAN	28.1	4.4

(B)

Query= FVC2

Length=386

Sequences producing significant alignments:	Score (Bits)	E Value
tr|B4DLN1|B4DLN1_HUMAN cDNA FLJ60124, highly similar to Mitocho...	53.9	2e-007
sp|P52815|RM12_HUMAN 39S ribosomal protein L12, mitochondrial O...	49.3	1e-006
sp|O60244|MED14_HUMAN Mediator of RNA polymerase II transcripti...	33.5	0.62
tr|H3BQK9|H3BQK9_HUMAN Microtubule-actin cross-linking factor 1...	33.1	0.82
tr|H3BPE1|H3BPE1_HUMAN Microtubule-actin cross-linking factor 1...	33.1	0.83
sp|Q9UL36|ZN236_HUMAN Zinc finger protein 236 OS=Homo sapiens O...	31.2	2.8
tr|J9JID5|J9JID5_HUMAN Zinc finger protein 236 OS=Homo sapiens ...	31.2	3.1
tr|H3BP71|H3BP71_HUMAN E3 ubiquitin protein ligase OS=Homo sapi...	30.4	4.9
tr|A0A804HIB7|A0A804HIB7_HUMAN Alstrom syndrome protein 1 (Frag...	30.4	5.3
sp|O75150|BRE1B_HUMAN E3 ubiquitin-protein ligase BRE1B OS=Homo...	30.4	5.5
tr|A0A804HKP4|A0A804HKP4_HUMAN Alstrom syndrome protein 1 (Frag...	30.0	7.6
tr|H3BUR1|H3BUR1_HUMAN Protein 4.2 (Fragment) OS=Homo sapiens O...	28.5	8.5
tr|A0A2R8Y4P4|A0A2R8Y4P4_HUMAN Transcription activator BRG1 OS=...	29.6	8.5
tr|A0A2R8Y7S2|A0A2R8Y7S2_HUMAN Transcription activator BRG1 OS=...	29.6	8.6

(C)

Sequences producing significant alignments:	Score (Bits)	E Value
sp|Q32AF8|RL7_SHIDS 50S ribosomal protein L7/L12 OS=Shigella dy...	228	5.00E-74
sp|Q31U11|RL7_SHIBS 50S ribosomal protein L7/L12 OS=Shigella bo...	228	5.00E-74
tr|F1XJ52|F1XJ52_ECO57 50S ribosomal protein L7/L12 OS=Escheric...	228	5.00E-74
sp|P0A7K5|RL7_SHIFL 50S ribosomal protein L7/L12 OS=Shigella fl...	228	5.00E-74
sp|P0A7K5|RL7_SHIFL 50S ribosomal protein L7/L12 OS=Shigella fl...	228	5.00E-74
sp|A8AKU0|RL7_CITK8 50S ribosomal protein L7/L12 OS=Citrobacter...	226	4.00E-73
sp|A6TGN9|RL7_KLEP7 50S ribosomal protein L7/L12 OS=Klebsiella ...	226	6.00E-73
tr|D0ZQV3|D0ZQV3_SALT1 50S ribosomal protein L7/L12 OS=Salmonel...	224	3.00E-72

(D)

Serial No.	Epitope Name	Sequence	Method	Result	Score
1	E1	LRLTPRPVS	SVM	POSITIVE	0.31856275
2	E2	VLQWASLAV	SVM	POSITIVE	0.16185077
3	E3	VVRAQGLDV	SVM	POSITIVE	0.5550728
4	E4	WASLAVLTL	SVM	POSITIVE	0.1626063
5	E5	VTFTSPPVT	SVM	NEGATIVE	-0.80908108

Figure S2: (A) Druggability blast of the vaccine candidate. (B) homology analysis of the vaccine candidate with gut microbiota. (C) homology analysis of the vaccine candidate with the human host. (D) IFN-gamma stimulation potential of the MHC-II-restricted epitopes.

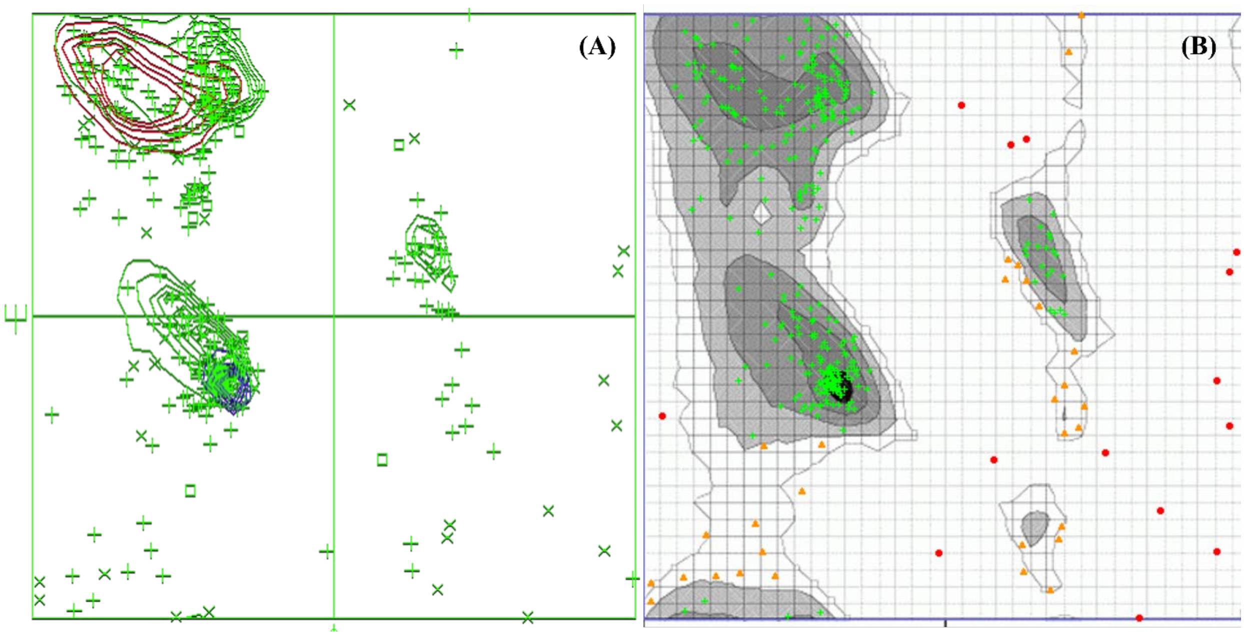

Figure S3: (A) Ramachandran plot computed using the WHAT IF server illustrating the most preferred region amino acids as +, and preferred region amino acids as ×; (B) Ramachandran plot computed using the Z-lab server showing green residues in the most preferred regions, yellow in the preferred regions, and red in the questionable regions.

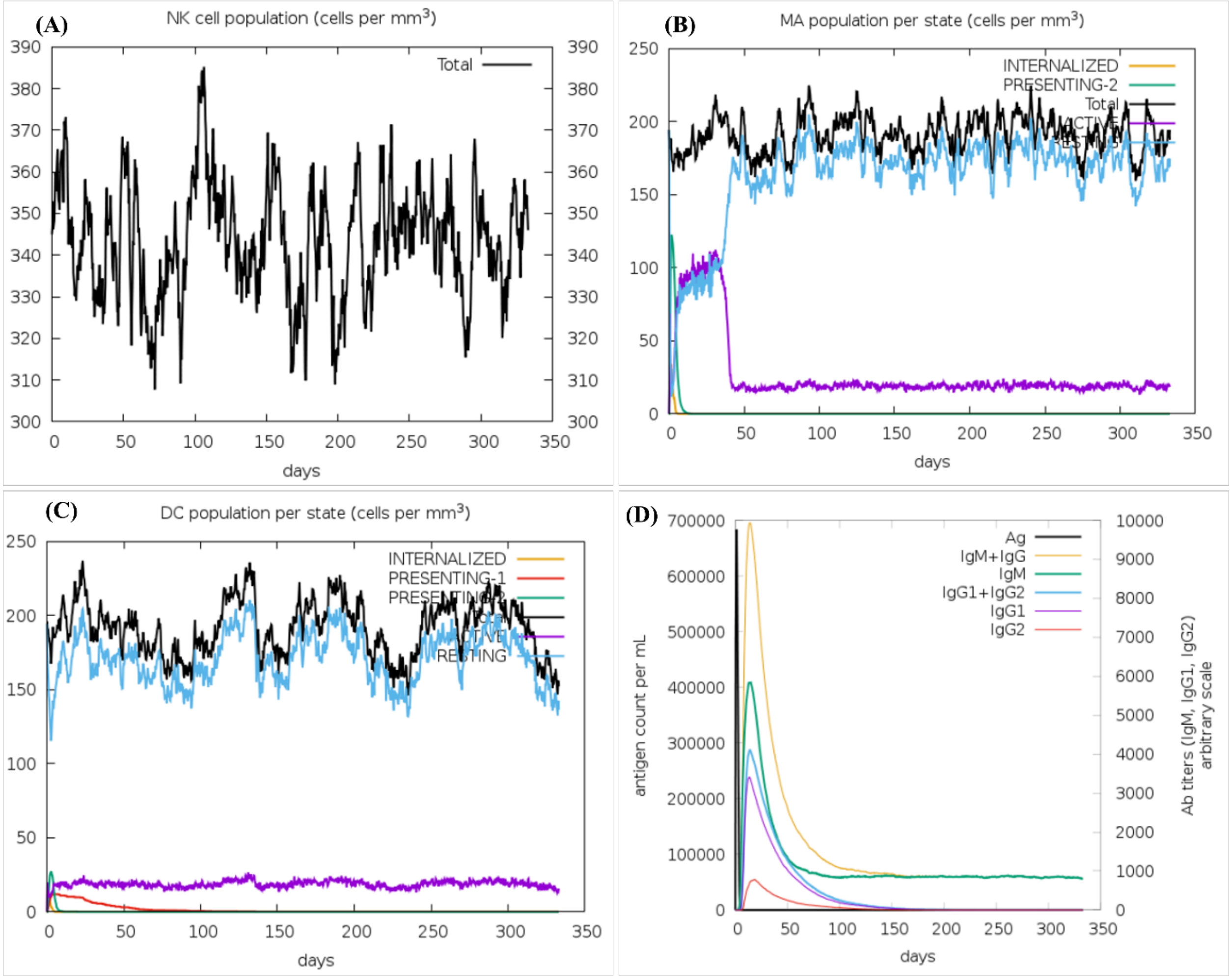

Figure S4: (A) NK cell population; (B) Macrophage (MA) cell population; (C) Dendritic cell (DC) population; (D) Antigen and Immune complex stimulation.

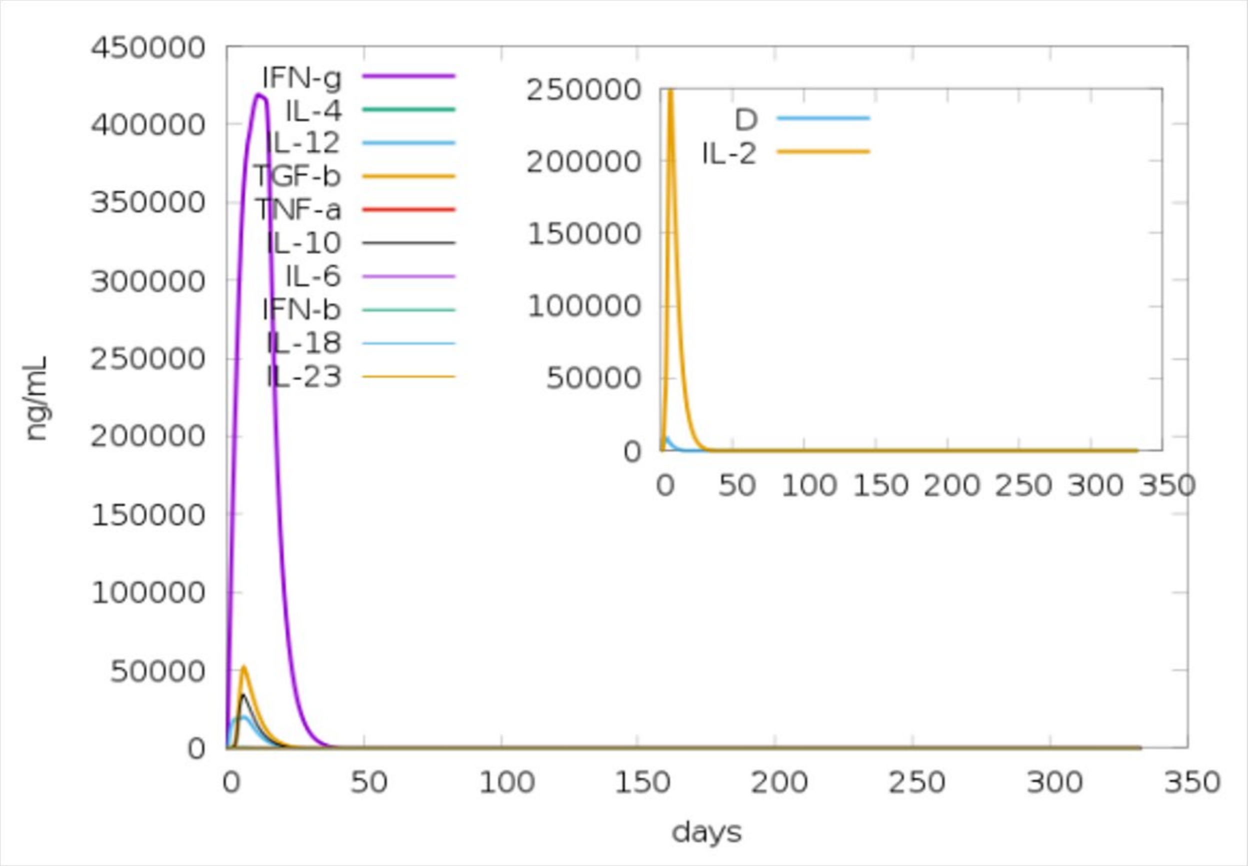

Figure S5: Danger signal and Cytokine stimulation.

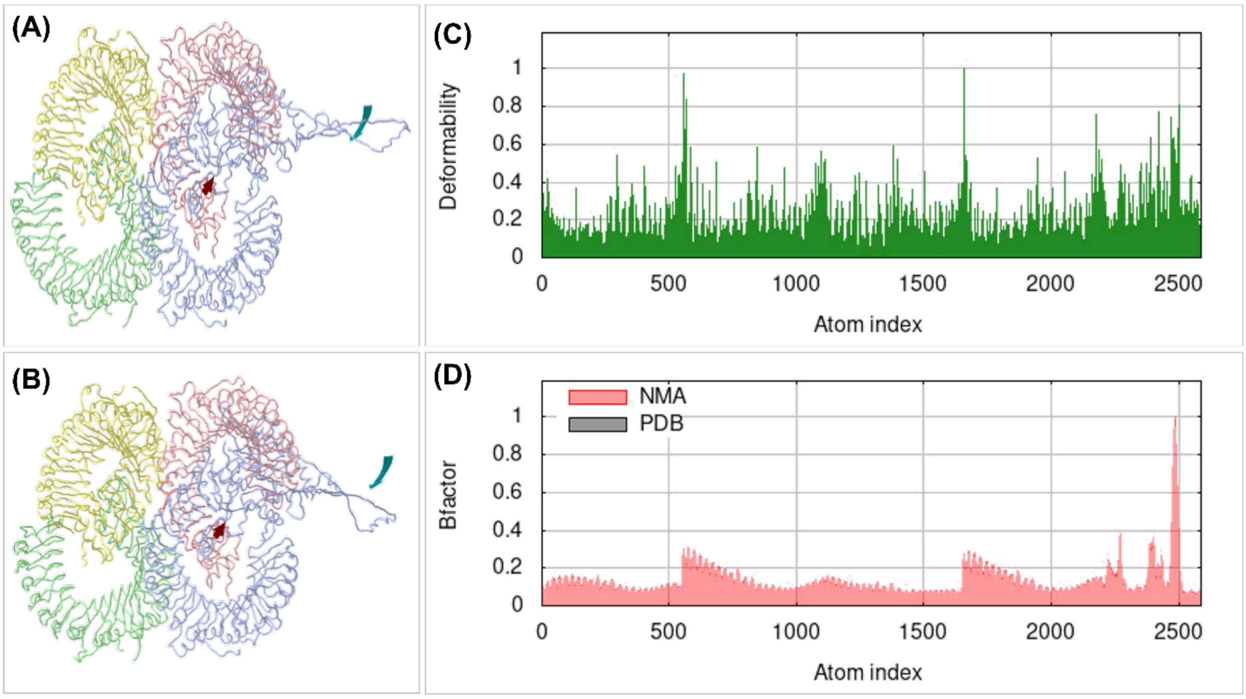

Figure S6: Molecular dynamics simulation of the docked complex. (A) MD simulation in the initial mode; (B) MD simulation in the final mode. A slight deformability is seen in the vaccine candidate with the green arrow showing downward movement and the red arrow showing upward movement of the residues; (C) deformability graph of the complex; (D) B-factor plot.

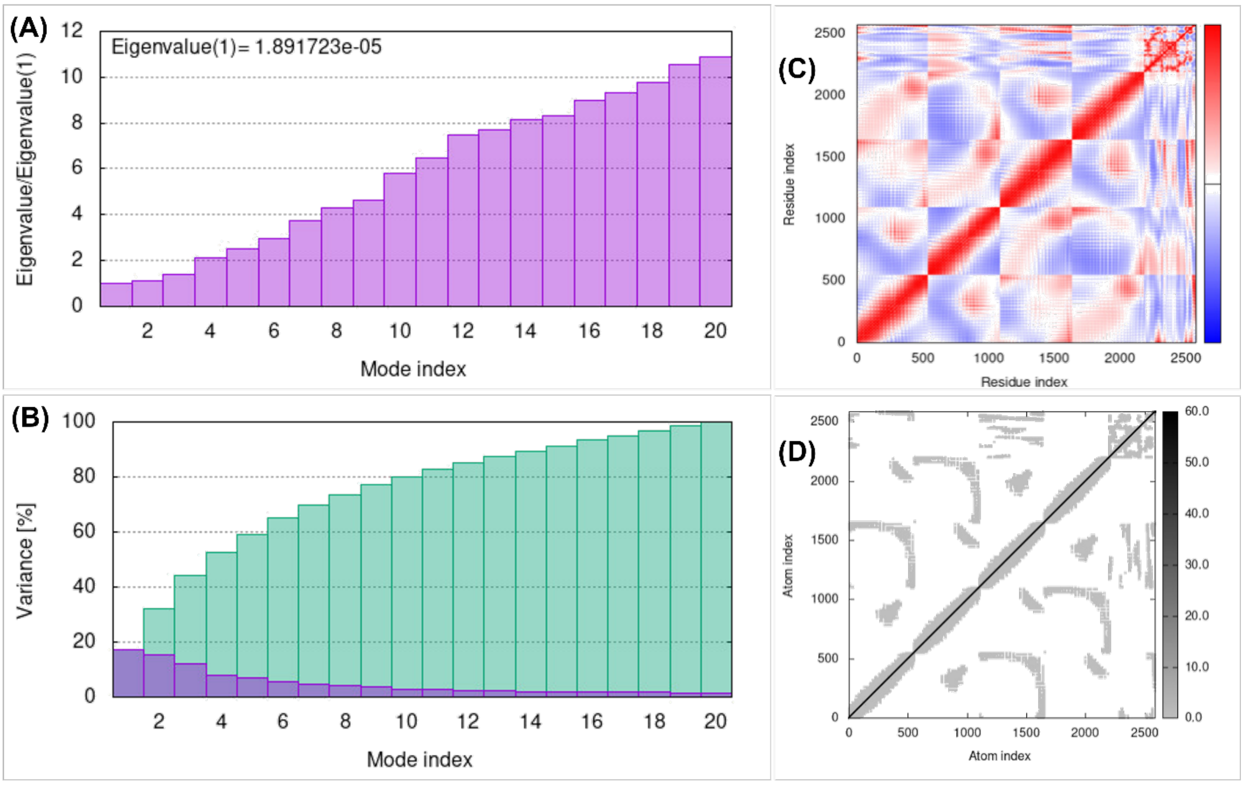

Figure S7: (A) eigenvalue plot. (B) variance % plot. (C) the covariance map; (D) the elastic network map of the docked complex

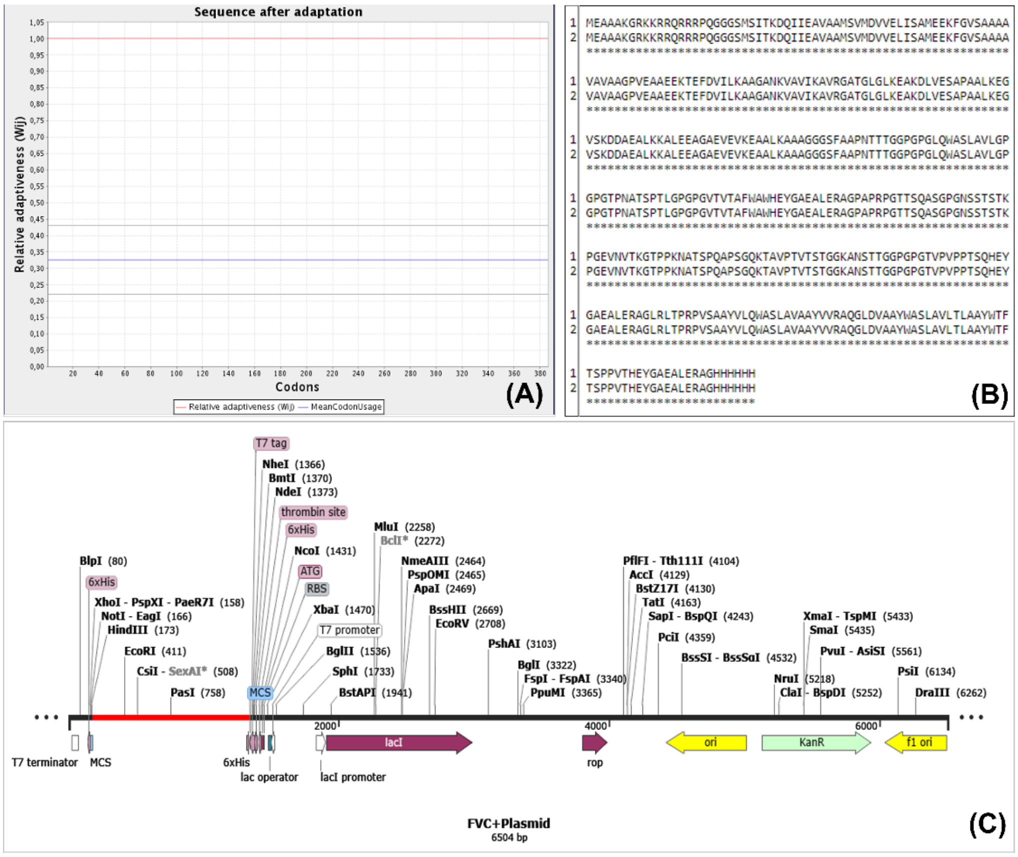

Figure S8: Expression analysis of the vaccine construct. (A) optimized codons with CAI 1.000 showing the maximum optimization; (B) CLUSTAL-W analysis showing 100% similarity between the pre-optimization (depicted as 1) and post-optimization (depicted as 2) vaccine constructs; (C) the linear map of cloned plasmid.

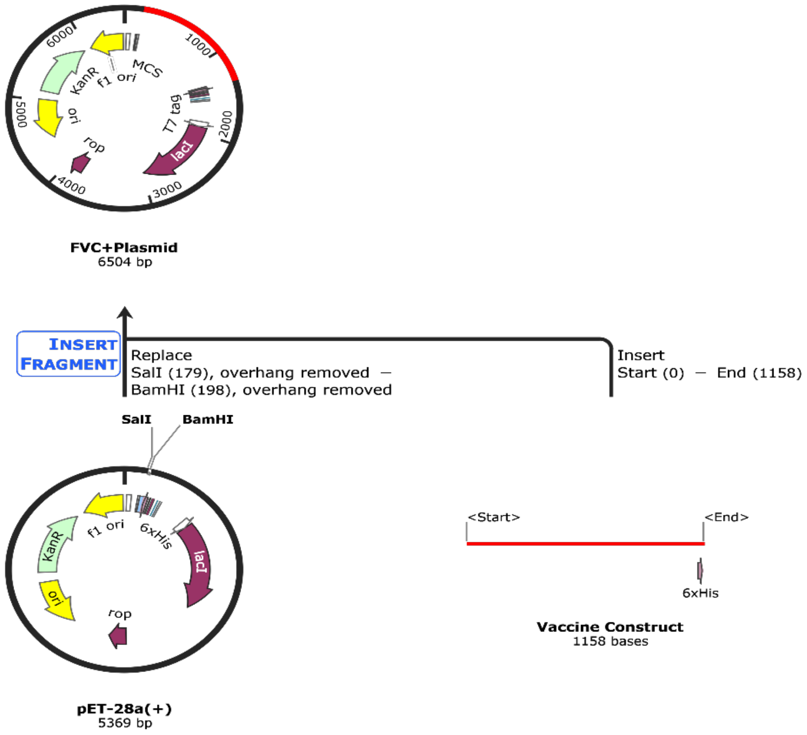

Figure S9: The history of *insilico* cloning.
